# New Data on Cylindrospermopsin Toxicity

**DOI:** 10.3390/toxins13010041

**Published:** 2021-01-08

**Authors:** Mariela Chichova, Oskan Tasinov, Milena Shkodrova, Milena Mishonova, Iliyana Sazdova, Bilyana Ilieva, Dilyana Doncheva-Stoimenova, Yoana Kiselova-Kaneva, Neli Raikova, Blagoy Uzunov, Diana Ivanova, Hristo Gagov

**Affiliations:** 1 Department of Animal and Human Physiology, Faculty of Biology, Sofia University “St. Kliment Ohridski”, 8 Dragan Tzankov blvd., 1164 Sofia, Bulgaria; mchichova@biofac.uni-sofia.bg (M.C.); mshkodrova@biofac.uni-sofia.bg (M.S.); mmishonova@biofac.uni-sofia.bg (M.M.); i.sazdova@biofac.uni-sofia.bg (I.S.); b.ilieva@biofac.uni-sofia.bg (B.I.); donchevast@biofac.uni-sofia.bg (D.D.-S.); neliraikova@biofac.uni-sofia.bg (N.R.); 2 Department of Biochemistry, Molecular Medicine and Nutrigenomics, Faculty of Pharmacy, Medical University of Varna, 55 Marin Drinov, 9002 Varna, Bulgaria; oskan.tasinov@mu-varna.bg (O.T.); yoana.kiselova@mu-varna.bg (Y.K.-K.); divanova@mu-varna.bg (D.I.); 3 Department of Botany, Faculty of Biology, Sofia University “St. Kliment Ohridski”, 8 Dragan Tzankov blvd., 1164 Sofia, Bulgaria; buzunov@uni-sofia.bg

**Keywords:** cylindrospermopsin, rat, liver, heart, mitochondria, semicarbazide-sensitive amine oxidase, SSAO, HIEC-6

## Abstract

Cylindrospermopsin (CYN) is a widely spread cyanotoxin that can occur in fresh water and food. This research aims to investigate CYN toxicity by studying the effects of drinking 0.25 nM of CYN-contaminated water from a natural source, and of the direct application of moderate concentrations of CYN on different animal targets. The chosen structures and activities are rat mitochondria inner membrane permeability, mitochondrial ATP synthase (ATPase) and rat liver diamine oxidase (DAO) activities (EC 1.4.3.22.), the force of the contraction of an excised frog heart preparation with functional innervation, and the viability of a human intestinal epithelial cell line (HIEC-6). The oral exposure to CYN decreased the reverse (hydrolase) activity of rat liver ATPase whereas its short-term, in vitro application was without significant effect on this organelle, DAO activity, heart contractions, and their neuronal regulation. The application of CYN reduced HIEC-6 cells’ viability dose dependently. It was concluded that CYN is moderately toxic for the human intestinal epithelial cells, where the regeneration of the epithelial layer can be suppressed by CYN. This result suggests that CYN may provoke pathological changes in the human gastrointestinal tract.

## 1. Introduction

The frequency of cyanobacteria blooms has been increasing worldwide due to climatic and anthropogenic factors [1]. Cyanobacteria produce a large number of biologically active secondary metabolites, which are highly toxic to animals and humans. Cyanotoxins are a structurally diverse group, in which the most important are microcystins, nodularins, cylindrospermopsin (CYN), saxitoxin, anatoxin-a and their natural analogues [2,3,4,5,6,7,8]. They manifest several major types of toxicity and act mainly as cytotoxins, hepatotoxins, neurotoxins, cardiotoxins, and dermatoxins [2,9].

CYN is a stable tricyclic quinidine alkaloid, whose presence in freshwater pools often increases [10] due to eutrophication and seasonal dynamics. CYN has been classified mainly as a hepatotoxin [11]. The pleiotropic CYN targets are liver [1], kidney [11], heart, eye, lung, spleen, ovary, T lymphocytes, neutrophils and vascular endothelium ([1,11,12] and references therein). CYN is known to inhibit protein synthesis, an effect observed also in its subtoxic concentrations [13]. CYN decreases both messenger RNA translation [13] and the expression of genes involved in ribosome biogenesis [14]. In primary cultures of rat hepatocytes, CYN inhibits glutathione synthesis [15]. CYN may induce oxidative stress either directly [11] or indirectly by the reduction of the glutathione formation [16]. Many studies on the toxic effects of CYN reported a decrease in cell viability and damage to cell organelles, including mitochondria [17].

Guideline values (GVs) for CYN in drinking water are in the range of 0.5–3 μg/L in different countries. Thus, in the United States the health advisory for infants is 0.7 μg/L (1.7 nM), and for children and adults, 3 μg/L (7.2 nM); the GV in Brazil is 15 μg/L (36 nM), and 20 μg/L (48 nM) in Ohio State, United States [18]. Global scanning of CYN indicates that its aquatic occurrence often exceeds not only the lowest GV accepted for drinking water—0.5 μg/L or approximately 1.2 nM, and 1 μg/L or 2.4 nM for recreational water—but also the highest GV of 20 μg/L for both drinking and recreational water, especially in the Asia–Pacific region, in reservoirs and rivers [18]. The amount of CYN in whole water samples varies from 0.00173 μg/L (4 pM) to 815 μg/L (2 μM) ([18] and references therein). CYN is accumulated in benthic biomass (up to 1580 μg/g) and pelagic biomass (up to 917 μg/g) [18]. Additionally, more than a 12-fold difference in bioaccumulation of CYN (0.00009 μg/g for *Oreochromis aureus* and 0.00126 μg/L *Heterandria jonesii*) for the same tissue [19] was observed in muscles from different fish species. All these data suggest the importance of studies on chronic intake of low doses of CYN, even if they are below the GV range, because this may reveal its specific and less pronounced species, tissue, and organelle effects.

Polyamines are important regulators of cell growth and cell death in health and disease [20]. Diamine oxidase (DAO) is an enzyme that catabolizes a variety of substrates including histamine and polyamines such as putrescine and spermidine in animals and humans. [21,22]. We hypothesized that the chronic application of low doses of CYN could alter DAO activity as another mechanism of its influence on inflammation and cell viability.

The common cytotoxic effects of CYN, such as inhibition of protein synthesis, oxidative stress, genotoxicity, and histopathological alterations [4], play a role in its neurotoxicity [12,23]. CYN could also cause neurological disorders through a more specific action on neuromediation [23]. The application of purified fractions isolated from the cyanobacteria *Cylindrospermopsis raciborskii* containing 10 μM CYN, inhibits the acetylcholine-activated inward current of snail neurons by 40% [24]. Similarly, CYN may influence the gut neurotransmission via acetylcholine signaling of the enteric division of the autonomic nervous system, which is a network of sensory neurons, motor neurons, and interneurons in the wall of the gastrointestinal tract. A short-time application of CYN on a functionally active frog heart preparation with preserved innervation in vitro might reveal new aspects of CYN influence, either directly on cardiomyocytes or indirectly via their nerve regulation.

The epithelial layer of the gastrointestinal tract is very important for the body’s general vulnerability to harmful compounds. It covers the large surface of the gastrointestinal tract, which is in contact with toxins in water and food, and is the common barrier against their absorption in inner-body fluids. Over the last two decades, the effect of CYN on the gastrointestinal tract has been tested using in vivo and in vitro experimental models (for details see [25]). A large amount of CYN-containing extract (2.5–8.3 mg/kg) was applied in the in vivo study [26]. It caused multiple ulcerations of the gastric mucosa as well as bleeding in the stomach and in the small intestine of mice. The median lethal dose was in the range 4.4–6.9 mg/kg [26]. CYN cytotoxicity in vitro was tested using Caco-2, an immortalized cell line of human colorectal adenocarcinoma cells [16,27,28]. CYN increased the paracellular apical-to-basolateral permeability of the pseudoepithelial layer [27,28], as well as the intracellular reactive oxygen species (ROS) concentration and glutathione content [16], but left the transcellular passage unchanged in Caco-2 [27].

The rising interest in CYN toxicity has led to intensive research in last two decades. However, some mechanisms and details of its toxicity remain to be elucidated. Additionally, unlike other cyanotoxins, studies into the effects of CYN on animals are limited [5]. Our preliminary investigation has shown the presence of CYN, and of a number of potential producers of CYN, in some Bulgarian waterbodies, including the largest Bulgarian coastal Lake Vaya [29]. However, the CYN producer there remains to be identified, because in the collected cyanoprokaryotes *Raphidiopsis raciborskii, Raphidiopsis mediterranea*, and *Chrysosporum bergii* the sulfotransferase gene, considered to be the best genetic marker for CYN-synthesizing strains were not detected [29].

The aims of this research are to study the toxicity of the chronic use of low concentrations of CYN-contaminated water from a natural source, and the effects of short-term application of moderate concentrations of CYN on different animal targets. They include rat mitochondria inner membrane permeability and mitochondrial ATP synthase (ATPase) activity, rat liver diamine oxidase (DAO) (EC 1.4.3.22.), an in vitro model of excitable tissues containing functionally active heart muscle cells with preserved innervation, and a continuously growing human intestinal epithelial cell line (HIEC-6).

## 2. Results

### 2.1. The Effect of Chronic Intake of Contaminated Water on Rat Liver Mitochondria

**Figure 1:**
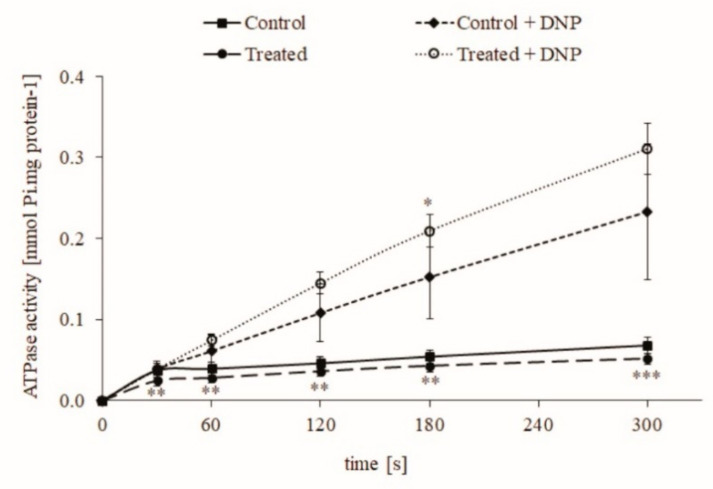
ATP synthase (ATPase) activity of intact rat liver mitochondria from untreated animals (Control) and animals with chronic intake of water from Lake Vaya (Treated). 2.4-dinitrophenol (DNP). Data are presented as mean ± standard error (SEM) of six treated animals. * < 0.05, ** < 0.01, and *** < 0.001—significant differences from the controls.

Mitochondria isolated from the control group and from rats that drank contaminated water (0.1 μg/L or 0.25 nM CYN) from Lake Vaya [30] for 24–28 days (treated group) showed lower permeability of mitochondrial membranes. Their ATPase activity remained low until the end of the measurements (Figure 1). The addition of the uncoupler 2.4-dinitrophenol (DNP) to the final concentration of 50 μM powerfully stimulated the ATPase reaction, which indicates a normal, functional state of mitochondria and a low permeability of the inner mitochondrial membranes isolated from both groups.

### 2.2. Effect of CYN on Freeze-Thawed Mitochondria

**Figure 2:**
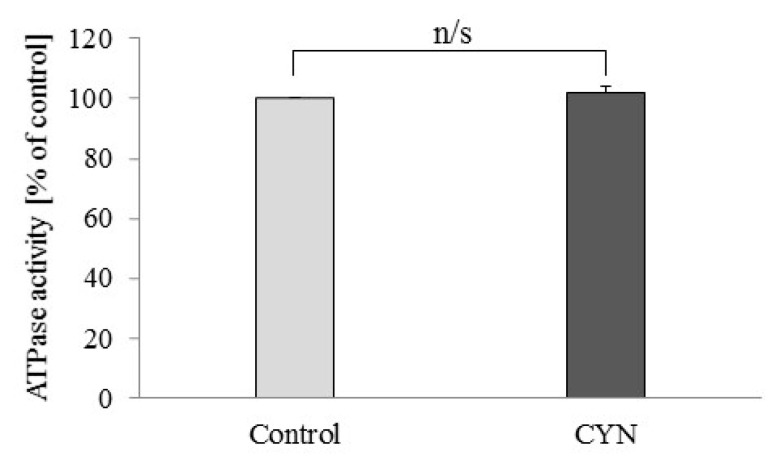
Effect of cylindrospermopsin (CYN) (1 μM) on ATPase activity of rat liver mitochondria destroyed by freezing and thawing. ATPase activity was calculated as a percentage versus the control group. Data are presented as mean ± SEM.

The application of 1 μM CYN did not change the ATPase activity of freeze-thawed mitochondria (Figure 2). The ATPase activity in the samples treated with CYN was 101.66 ± 2.11% of the control activity.

### 2.3. DAO Activity

**Figure 3:**
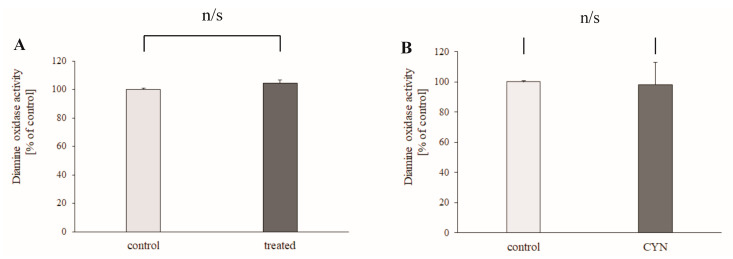
(**A**) Rat liver diamine oxidase (DAO) activity after 24–28 days of drinking contaminated water. (**B**) Direct effect of application of CYN (1 μM) on DAO activity. DAO activity values were calculated as percentages of the enzyme activity of the controls. Data are plotted as mean ± SEM.

The experiment on the effect of chronic intake of contaminated water on rat liver DAO activity demonstrated that drinking of contaminated water from Lake Vaya did not change significantly the activity of DAO (4.9% ± 2.53%, *p* = 0.236, n = 6) (Figure 3A). The direct effect of CYN (1 μM) was also studied. In the presence of CYN, the activity of DAO was 101.95 ± 9.87% of the control (*p* = 0.686, n = 4) (Figure 3B).

### 2.4. Effect of CYN on Frog Heart Preparations In Vitro

**Figure 4:**
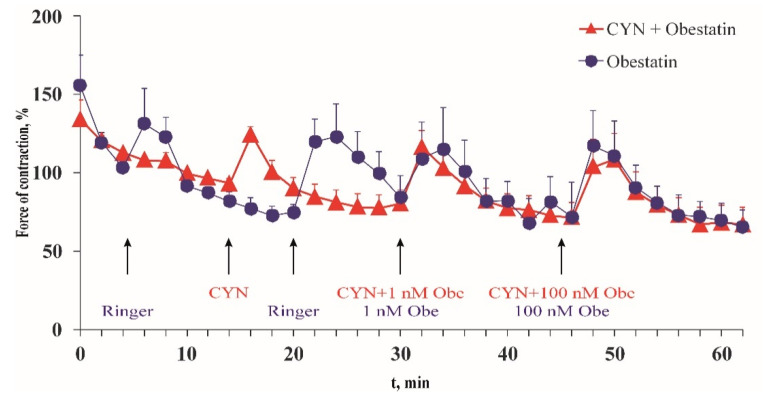
CYN (1 μM) (▲) effect on the maximal force of contractions of excised frog hearts. The effect of obestatin in the presence of CYN was compared with that of the obestatin-containing solution (●). The maximal force of contractions was calculated as a percentage of the force of contractions, measured at the 10th min from the start of the experiment that was taken as 100%. Data are expressed as means ± SEM of six experiments.

In the presence of 1 μM CYN, the force and frequency of the frog heart contractions were statistically insignificant when compared with the time control (Table 1). In the presence of CYN, the application of obestatin, an activator of epinephrine secretion from the sympathetic nerves in the heart wall, increased the force of the heart contractions (Figure 4). The maximum amplitude of the force of contractions in the presence of CYN was 125% ± 4.74% of the initial value, while in the control experiments it was 134% ± 22.7%.

The maximal positive inotropic effect was observed 4 min after the administration of 1 nM obestatin, and 2 min after administering obestatin together with 1 μM CYN. The strength of the heart contractions increased by 28% compared with the control group at the same time. The higher obestatin concentration (100 nM) in the presence of the toxin enhanced the amplitude of the contraction by 31%. In the absence of the toxin, obestatin showed a commensurate positive inotropic effect (26% and 40% for 1 nM and 100 nM, respectively).

### 2.5. CYN Cytotoxicity Tested on HIEC-6

**Figure 5:**
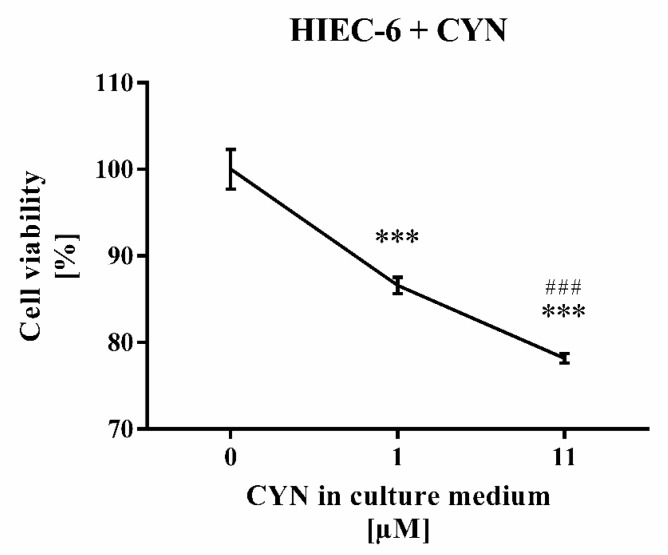
Treatment of human intestinal epithelial cell line (HIEC-6) cells with 1 µM or 11 µM CYN for 24 h. Each concentration was applied in two replicates vs. three controls. Data are presented as mean ± SD. Legend: *** *p* < 0.001 vs. untreated cells; ^###^ *p* < 0.001 vs. 1 µM CYN treatment.

Treatment of human intestinal epithelial cell line (HIEC-6) with higher CYN concentration (11 µM) reduced stronger the cell viability (by 21.8%, *p* < 0.001) than treatment with its lower (1 µM) concentration (by 13.4%, *p* < 0.001) (Figure 5).

## 3. Discussion

Mitochondria are the common cell source of ATP needed for processes like transmembrane transport, muscle contraction, synthesis, secretion, and intracellular signaling. Drinking water contaminated with CYN for weeks could harm oxidative phosphorylation in rat mitochondria by affecting their protein synthesis, cytochrome 450, and inner membrane permeability [2]. The rat is a well-studied experimental animal model that is widely used in various types of toxicological studies. On the other hand, rats inhabit polluted areas and have stronger adaptive potential against harmful contaminators when compared with other animals and humans, as well as powerful antioxidant systems, and the ability to synthesize vitamin C [31].

According to our knowledge, the effect of CYN on mitochondrial ATP synthase activity has not been studied. For this reason, we undertook a study of the effect of CYN on ATPase activity in rat liver mitochondria. As a result, the drinking of CYN-contaminated water significantly decreased the reverse mode of this enzyme, i.e., the ability of ATPase of rat liver mitochondria to hydrolyze ATP. This result suggests that chronic treatment with a low concentration of the toxin increases oxidation processes and pH in a mitochondrial matrix that can hyperpolarize the inner mitochondrial membrane. Under physiological conditions, this effect will enhance ATP synthesis [32]. In the presence of the uncoupler 2.4-dinitrophenol, the hydrolase activity of ATPase from treated animals was similar to that of the controls, which suggests that the amount and maximal activity of the enzyme are similar. There are not enough data to explain the mechanism of these data obtained in animals treated with low CYN-contaminated water. It could be supposed that low doses of CYN have a hormesis effect on rat liver mitochondria. On the other hand, the short-term application of 1 µM CYN in vitro did not change the activity of mitochondrial ATPase, i.e., there is not a direct effect of a low concentration of CYN on this key enzyme of cellular energetics.

The liver is the primary organ responsible for the metabolism of toxic xenobiotics, but it is also very sensitive to their toxicity. CYN has initially been classified as a hepatotoxin [12]. This has been confirmed both in vitro in primary rodent hepatocytes and human liver hepatocellular carcinoma (HepG2) cells [12] and references therein, and in rodent models in vivo [26,33]. Additionally, non-cytotoxic concentrations of CYN increase ROS production, which most probably is related to decreased glutathione synthesis [16]. Polyamines, the common substrate of DAO, are important regulators of cell growth and viability [20]. For these reasons, the activity of DAO, an important liver enzyme that deaminates short-chain primary amines, was studied under conditions similar to those for mitochondria: contaminated drinking water from Lake Vaya was given to a treated group and CYN was applied directly to the DAO-containing fraction. In both cases, a significant effect of CYN was not observed. It is concluded that neither the drinking of low CYN-containing water, nor the application of a moderate concentration of CYN, influences rat liver DAO activity.

Excised frog heart preparations were used in another set of experiments. This in vitro model exhibits physiologically active, autonomic nerve projections and muscle cells (Ilieva, Gagov, Sazdova, unpublished data). The preparation is very useful for pharmacological and toxicological studies on the neural regulation of heart muscle contractions, because it provides both tissues with preserved excitability, metabolism, and intracellular signaling. Under our experimental conditions, short-term application of CYN (1 µM) did not affect the force or the frequency of spontaneous contractions of the excised frog hearts. This result is in agreement with previous studies on mice where high doses of CYN in vivo caused a delayed effect only: occasional single heart cell necrosis 48 h after intraperitoneal injection of 0.2 mg/kg [33] or myocardial hemorrhages [27] 48 h after gavage administration of 2.5–8.3 mg/kg. Obestatin, an indirect activator of heart muscle [34], retained its positive inotropic effect in the presence of the toxin. Obestatin acts on heart muscle tissue by increasing the release of epinephrine from the varicosities of autonomic axons. CYN presence had no effect on obestatin’s regulatory influence on heart contractions, i.e., 1 µM CYN does not have a rapid neurotoxic or modulatory effect (30 min) when it is applied directly to heart muscle with functional sympathetic neuronal projections.

CYN cytotoxicity was tested for the first time on continuously growing HIEC-6 cells that expressed functional markers for human intestinal crypt cells. The number of viable cells was significantly reduced in the presence of 1 µM CYN and even more so at 11 µM CYN. This result differs from that with Caco-2 cells, where no noticeable effect on viability was observed in the presence of 10 µM CYN measured by AlamarBlue assay [28], or of 20 µM CYN determined by exclusion of Trypan Blue [27]. On the other hand, exposure of Caco-2 cells to CYN for 48 h reduced their protein content depending on concentration, which was significantly above 2.5 µg/mL CYN, and increased over time (24 h vs. 48 h) [16]. These data support the view that CYN sensitivity of gastrointestinal epithelium continuously decreased aborally (ileum > colon) as suggested in [35]. The molecular mechanisms of this CYN cytotoxicity most probably include an inhibitory effect on ribosomal gene expression and ribosome biogenesis, decreased protein synthesis, enhanced ROS production, and genotoxicity of cytochrome P450-generated metabolites [4,14,16,36].

It can be concluded that CYN is toxic to human epithelial cells of the gastrointestinal tract, where the intensive regeneration of the epithelial layer could be suppressed in the presence of low doses of CYN. This result suggests that intestinal epithelial cells, cells which are continuously dividing, are very sensitive to CYN. The presence of CYN, especially if it is in combination with another toxin [10] or an environmental factor that causes stress, may induce pathological changes in the human gastrointestinal tract. Moreover, the intestinal epithelial layer is located on the mucosal border and for this reason it is not as well protected by the body homeostasis as the inner tissues. Concerning mitochondria, it is suggested that only higher doses of CYN can affect them, probably by a protein synthesis inhibition and oxidative stress [11,17]. Prolonged treatment with contaminated water, containing CYN below the GV for drinking water, might support the oxidative processes and increase the pH gradient as suggested by its anti-uncoupling effect in rat liver mitochondria. Future studies are needed to reveal details of the CYN effect on these cellular organelles.

## 4. Materials and Methods

### 4.1. Isolation of Intact Rat Liver Mitochondria

Intact liver mitochondria were isolated from mature Wistar rats using the method of Johnson and Lardy [37], with modifications [38]. All experimental procedures were conducted in accordance with the Guiding Principles for Care and Use of Laboratory Animals approved by the Bulgarian Center for Bioethics and are in accordance with the International Guiding Principles for Biomedical Research Involving Animals. We have ethical approval with code (No.) 224 from 23.01.2019, issued by Bulgarian Food Safety Agency, Ministry of Agricultural, Food and Forestry. Rats were anesthetized by inhalation of diethyl ether and then decapitated.

### 4.2. Assay of Mitochondrial ATPase Activity

ATPase activity of mitochondria was determined by measuring the inorganic phosphate (Pi) released from ATP as stated in [38]. The ATPase reaction was carried out in an assay medium consisting of 200 mM sucrose, 10 mM KCl, 50 mM Tris-HCl, 100 μM EDTA-KOH, and 1 mM ATP (pH 7.5). A total of 50 µM 2.4-dinitrophenol and 1 µM CYN were included wherever indicated. The amount of Pi was measured spectrophotometrically.

### 4.3. ATPase Activity of Intact Rat Liver Mitochondria

Six healthy Wistar rats (200–250 g in weight) were subjected to chronic *ad libitum* intake of water from Lake Vaya for 24–28 days. Experiments with intact (coupled) mitochondria were performed to study the effect of contaminated water on the membrane permeability of mitochondria. For this purpose, after the expiration of the treatment period in the three consecutive experiments, each of which included two treated and one control animal, intact liver mitochondria were isolated by differential centrifugation and their ATPase activity was determined and expressed in μM Pi/mg protein. Some of the mitochondrial suspensions obtained from the control animals were frozen and used in the next series of experiments.

### 4.4. ATPase Activity of Freeze-Thawed Mitochondria

In a second group of in vitro experiments, the direct effect of the toxin CYN on the ATPase activity of freeze-thawed mitochondria from control animals was investigated. Freezing and subsequent thawing leads to disruption of the inner mitochondrial membrane. ATPase activity was recorded in medium with the same composition. The toxin was administered at a final concentration of 1 μM, and after preincubation with the mitochondrial suspension for 10 min, the reaction was started by adding ATP at a final concentration of 1 mM. ATPase activity was calculated as a percentage of the activity (expressed in μM Pi/mg protein min) measured under control conditions (without added CYN).

### 4.5. Assay of Rat Liver DAO Activity

Rats for in vivo experiments were used after a 25-day treatment and compared to untreated control animals of the same age. The liver was quickly isolated, washed with cold sodium phosphate buffer 0.01M, pH 7.0, weighed, and homogenized in a ratio of 1:4 (w:v) with the same buffer. The homogenate was heated at 60 °C in a water bath for 10 min and centrifuged at 20,000× *g* for 20 min. The supernatant was collected for analysis. DAO activity was determined spectrophotometrically using putrescine as a substrate [39]. Hydrogen peroxide, formed in the amine oxidase reaction, formed a color complex with phenol and 4-aminoantipirine in the presence of peroxidase. The extinction was measured spectrophotometrically at λ = 500 nm. Protein content was determined by the method of Lowry [40], with bovine serum albumin as a standard.

The standard reaction mixture for the DAO activity assay (3.0 mL final volume), before the photometric measurement, contained 0.1 M sodium phosphate buffer (pH 7.4), 0.82 mM 4-aminoamtipirine, 10.6 mM phenol, 4 IU/mL of horseradish peroxidase, 2.5 mM putrescine, 1.0 mM semicarbazide, and 300 µL of supernatant.

The blanks, containing a buffer, an enzyme source, and peroxidase, were preincubated with semicarbazide at 37 °C for 20 min. The control samples contained the same components, except semicarbazide. The blanks and the samples also contained CYN (1 μM). After preincubation, putrescine, 4-aminoantipirin, and phenol were added, and all tubes were incubated at 37 °C for 60 min. The reaction was stopped by chilling the tubes on ice and semicarbazide was added to the samples.

### 4.6. Study of Excised Frog Heart Contraction

*Pelophylax ridibundus* frogs were denervated by double pithing and the hearts were cannulated and isolated by the modified Straub method [41]. Contractions were registered by the highly sensitive force transducer GRASS. Data were recorded and analyzed by the software TENZSU and TENZOGRAPH (Stocks, Sofia, Bulgaria). For a more detailed description see [34]. In excised heart preparations, the sympathetic nerve projections remain functional (Ilieva, Gagov, Sazdova, unpublished data). This allows for the testing of the effect of CYN on two excitable structures simultaneously—the heart muscle and the adrenergic axonal projections.

All experiments were performed at room temperature (20–22 °C). In the control group (n = 6), cardiac activity was measured and Ringer solution (200 μL) in the cannula was replaced every 15 min. Excised frog heart preparations developed regular contractions with stable patterns and force. Under our experimental conditions, the spontaneous contractions slightly declined during the experiment (16% on average), except for the first 10 min of the experiment, when the initial moderate decrease was greater, at 48% on average. According to the Frank–Starling mechanism, each application of a fresh solution causes a short-term increase in heart contractions, which was always observed, regardless of the solution’s composition. In the second experimental group, after 15 min of adaptation, we applied CYN at a concentration of 1 μM and obestatin (1 and 100 nM) in the presence of CYN.

### 4.7. Cytotoxicity Test

HIEC-6 was purchased from the American Type Culture Collection (ATCC^®^). Cells were cultured according to the manufacturer’s protocol using 75 cm^2^ flasks at 37 °C in a humidified chamber with a 5% CO_2_ atmosphere. Complete growth medium containing OptiMEM 1 Reduced Serum Medium (Gibco) supplemented with 20 mM HEPES, 10 mM GlutaMAX (Gibco), 10 ng/mL Epidermal Growth Factor, fetal bovine serum (Sigma-Aldrich) at a final concentration of 4%, and a penicillin/streptomycin mixture to final concentrations of 1% was used.

HIEC-6 cells were seeded in 12 well flasks with 1 mL of complete growth medium at a density of 6.5 × 10^4^ cells/well and let to adhere overnight. After 24 h, the culture medium was replaced and cells were treated for an additional 24 h with 1 µM and 11 µM CYN (Sigma Aldrich, HPLC standard, water solution) dissolved in a complete growth medium. Before dissolving in the growth medium, the volume of the sample with 11 µM CYN was reduced using vacuum centrifuge, to obtain an equal volume of both concentrations. Each concentration was applied in two replicates, and the control treatment in triplicate.

The viability of treated cells was estimated using 3-(4.5-dimethylthiazol-2-yl)-2.5-diphenyltetrazolium bromide (MTT, AppliChem) [42]. The assay is based on the ability of viable cells to reduce MTT to purple insoluble formazan crystals. At each well, 100 μL of MTT solution (2 mg/mL) dissolved in phosphate-buffered saline (pH = 7.4) was added 20 h after treating the cells. After 4 h of incubation, the medium was removed and 1 mL/well of dimethyl sulfoxide (Scharlau Chemie S.A.) was added for cell lysis. Absorbance was measured at a wavelength of 550 nm using a Synergy 2 plate reader (BioTek). Untreated cells were used as the control. Cell viability (%) was calculated as [(mean absorbance of the sample/mean absorbance of the control) × 100]. Results are presented as mean ± SD.

### 4.8. Statistical Analysis

Data are presented as mean ± standard error (SEM) usually from six samples. The difference between the treated samples and the untreated controls was tested by a one-way analysis of variance (ANOVA) assay. Statistical analyses of HIEC-6 cells data were performed using Graph Pad Prism V6.0 software. Values of *p* < 0.05 were considered as significant.

## Figures and Tables

**Table 1:** Time-dependent effect of CYN (1 μM) on the maximal force of frog heart contractions.

Time after Application	Time Control Ringer, ±SEM	CYN, ±SEM	*p*
2 min	93% ± 14.28% (*n* = 7)	125% ± 4.74% (*n* = 6)	0.091
4 min	90% ± 15.86% (*n* = 7)	101% ± 7.09% (*n* = 6)	0.552
6 min	75% ± 14.98% (*n* = 7)	90% ± 7.03% (*n* = 6)	0.388

## Data Availability

The data presented in this study are available on request from the corresponding author. The data are not publicly available due to them not having been published yet.
